# Metformin and its sulphonamide derivative simultaneously potentiateanti-cholinesterase activity of donepezil and inhibit beta-amyloid aggregation

**DOI:** 10.1080/14756366.2018.1499627

**Published:** 2018-09-24

**Authors:** Magdalena Markowicz-Piasecka, Kristiina M. Huttunen, Joanna Sikora

**Affiliations:** a Laboratory of Bioanalysis, Department of Pharmaceutical Chemistry, Drug Analysis and Radiopharmacy, Medical University of Lodz, Lodz, Poland;; b Faculty of Health Sciences, School Of Pharmacy, University of Eastern Finland, Kuopio, Finland

**Keywords:** Metformin, donepezil, Alzheimer’s disease, acetylcholinesterase, amyloid

## Abstract

The aim of this study was to assess *in vitro* the effects of sulphenamide and sulphonamide derivatives of metformin on the activity of human acetylcholinesterase (AChE) and butyrylcholinesterase (BuChE), establish the type of inhibition, and assess the potential synergism between biguanides and donepezil towards both cholinesterases (ChEs) and the effects on the β-amyloid aggregation. Sulphonamide **5** with *para-*trifluoromethyl- and *ortho-*nitro substituents in aromatic ring inhibited AChE in a mixed-type manner at micromolar concentrations (IC_50_ = 212.5 ± 48.3 µmol/L). The binary mixtures of donepezil and biguanides produce an anti-AChE effect, which was greater than either compound had alone. A combination of donepezil and sulphonamide **5** improved the IC_50_ value by 170 times. Compound **5** at 200 µmol/L inhibited Aβ aggregation by ∼20%. In conclusion, *para-*trifluoromethyl-*ortho-*nitro-benzenesulphonamide presents highly beneficial anti-AChE and anti-Aβ aggregation properties which could serve as a promising starting point for the design and development of novel biguanide-based candidates for AD treatment.

## Introduction

Alzheimer’s disease (AD) is a progressive neurodegenerative disease featured mostly in the form of dementia in the elderly. The aetiological features of AD include cerebral senile plaques (SPs) due to deposition of β-amyloid (Aβ), neurofibrillary tangles (NFTs) composed of tau hyperphosphorylation, and decreased level of acetylcholine (ACh)^1–4^. The global burden of the population suffering from AD was established to reach ∼44 million in 2015. According to Kumar et al.^5^ this number is expected to double by 2030 and triple by 2050^5^. Therefore, as the incidence of AD has increased year by year, prevention, control, and search for novel anti-AD therapeutics has become globally focused^5^.

Clinically, only symptomatic treatments including acetylcholinesterase inhibitors (AChEIs) such as donepezil, rivastigmine, and galantamine along with N-methyl-D-aspartate (NMDA) receptor antagonist (memantine) have been approved for the treatment of AD^6^^,^^7^. Nowadays many preclinical and clinical trials on novel drugs in the treatment of AD are undergoing, however, to this day successful cure with a single one-target drug therapy has failed due to the manifold pathophysiology of AD. During the last decade, new therapeutic approaches to AD treatment have been formulated on the basis of current neurobiological knowledge of the complex nature of AD^1^. One of the leading approaches in the drug design strategy relies on the synthesis of multi-target directed (MTD) ligands, which might be promising candidates for the treatment of multi-factorial AD^7^^,^^8^. So far, the development of such drugs has achieved some success in the improvement of cognitive functions in AD, whereas they have failed in several aspects of disease modification. Novel strategies include those that aim to reduce the formation of amyloid peptides by inhibiting the β-secretase and γ-secretase enzymes. Furthermore, immunotherapy has been developed for the purpose of inhibiting β-amyloid peptide aggregation^9^. Apart from the above-mentioned approaches, there are also trends in drug discovery in relation to cholinergic and monoaminergic systems and their effects on cellular energy metabolism^10^. Potential drug candidates are studied in relation to energy metabolism, mitochondrial functions, and production of reactive oxygen species (ROS)^10–13^. One of these agents might be metformin, an oral anti-diabetic drug, which does not only decrease the plasma glucose level in several mechanisms but has also been shown to exert anti-inflammatory, anti-apoptotic, and anti-oxidative properties^14^. Recently metformin has also been repurposed as a potential anticancer drug^15^.

**Figure 1.**
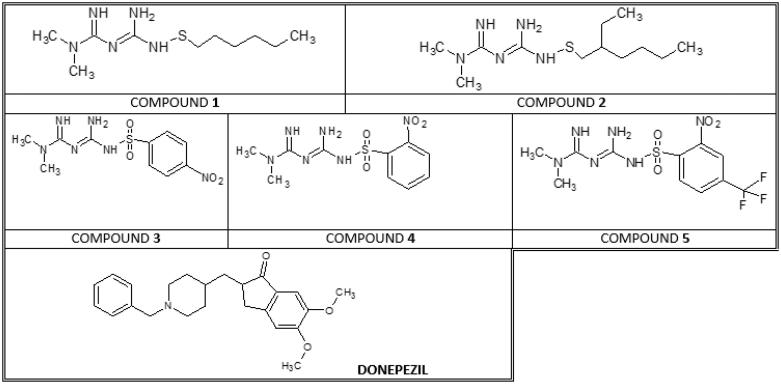
Chemical structure of tested biguanide derivatives – compounds **1**–**5** and donepezil. All compounds were prepared in form of hydrochlorides.

Currently available evidence suggests that metformin may play an important role in the treatment of AD, however, the available literature on metformin’s effects on the central nervous system and its potential role in AD treatment is limited and predominantly consists of *in vitro* studies and a few *in vivo* studies^16^. Some clinical studies confirm its beneficial effects regarding cognitive impairment and memory loss. The researchers also highlight the advantageous activity of metformin on cognitive performance in depressed patients with type 2 diabetes mellitus (T2DM)^17^^,^^18^. These advantageous activities of metformin might originate in its molecular mechanism of action. *In vitro*, the activation of AMPK (5′ adenosine monophosphate-activated protein kinase) by metformin has a neuroprotective effect on human neural stem cells, restores mitochondrial functions and weakens advanced glycation end products (AGEs) effects^19^. Furthermore, metformin protects against cytotoxic stress and mitochondria-mediated cell death and can improve insulin sensitivity in a neuronal cell line^20,21^. Some authors point out that metformin is responsible for the stimulation of neurogenesis in the mouse brain, and has positive effects on the vascular system, including brain endothelial cells^22^. In addition, the inhibitory properties of metformin on AChE, whose levels are elevated both in AD and T2DM should also be mentioned. Individual *in vivo* studies conducted on animals imply that chronic treatment with metformin might improve cognitive performance, reduce oxidative stress and ChE activity^23^^,^^24^. In our previous study^25^ it was reported that metformin inhibited 50% of the AChE activity at milimolar concentrations (2350 µmol/L), in a mixed inhibition manner and seemed to be selective towards AChE since it presented low anti-BuChE activity. Phenformin was shown to be less active towards AChE in comparison with metformin (IC_50_ = 4940 µmol/L), but it was also found to inhibit BuChE competitively (IC_50_ = 259.0 µmol/L)^25^. The obtained results suggest that biguanides might act as a novel class of inhibitors for AChE and BuChE and encourage us to undertake further studies for the development of both selective and non-selective inhibitors of ChEs. Therefore, the purpose of this paper was to explore *in vitro* the effects of two previously unstudied sulphenamides (N–S–) with varying numbers of carbon atoms in alkyl chain and a series of sulphonamides (N–SO_2_–) of metformin (Figure 1) on the activity of human AChE and BuChE, and to establish the type of inhibition. Furthermore, the potential synergism of selected biguanides and donepezil towards both cholinesterases (ChEs) was assessed. The final part presents an estimation of biguanides potential to inhibit beta-amyloid aggregation. The enclosed findings will provide a greater insight into the potential application of biguanide derivatives as effective adjuvants to clinically approved acetylcholinesterase inhibitors.

## Materials and methods

### Materials

Compounds **1**–**5** (Figure 1) were designed and synthesised at the University of Eastern Finland as reported elsewhere^26^^,^^27^. On the basis of estimated therapeutic plasma concentrations of metformin (0.8 nmol/mL–0.6 µmol/mL) and our previously conducted studies decided to study the compounds at the maximal concentration of 4 µmol/mL. The concentration of metformin, phenformin and derivative **5** for simultaneous testing with donepezil (Sigma Aldrich, Darmstadt, Germany) was chosen on the basis of their IC_50_ values and potential therapeutic concentration^28^^,^^29^.

The following reagents were used in the ChEs inhibition studies: 0.9% NaCl (0.15 mol/l) (Chempur, Tarnowskie Góry, Poland); 0.1 mol/l phosphate buffer pH = 7.0 and pH = 8.0 (disodium phosphate, monosodium phosphate (J.T. Baker, Center Valley, PA); a stock solution of 5,5′-dithiobisnitrobenzoic acid (DTNB; 0.01 mol/l (Sigma Aldrich, St. Louis, MO)) prepared in phosphate buffer at pH = 7.0; a stock aqueous solution of acetyltiocholine iodide (ATC) (21.67 mg/mL) (Sigma Aldrich, Darmstadt, Germany); a stock aqueous solution of butyryltiocholine iodide (BTC) (20.50 mg/mL) (Sigma Aldrich, Darmstadt, Germany). All solutions were stored as small samples at a temperature of –30 °C and before each experiment, were restored at 37° C for 15 min. For the establishment of kinetic parameters and the type of inhibition decreasing concentrations of ATC and BTC were used (1:2–1:20).

### Biological material

Blood samples were obtained from healthy donors from the Voievodal Specialized Hospital in Łódź, Poland (*Wojewódzki Specjalistyczny Szpital im. Dr W. Biegańskiego w Łodzi*). The blood was collected into vacuum tubes containing sodium citrate. Erythrocytes were separated from plasma by centrifugation (3000 × g, 10 min, 20 °C) with a Micro 22 R centrifuge (Hettich ZENTRIFUGEN, Tuttlingen, Germany) and washed three times with 0.9% saline. Afterwards, red cells were haemolysed by freezing and stored at a temperature of –30 °C; before each experiment, they were restored at 37° C for 15 min and used to determine AChE activity. Plasma for determination of BuChE activity was obtained by centrifuging the blood (3000 × g, 10 min, 20 °C). The studies on the biological material were approved by the Bioethics Committee of the Medical University of Lodz (RNN/27/18/KE).

### General cholinesterase inhibition

Before commencing the studies, probationary experiments between the reagents (DTNB, ATC, and BTC) and tested metformin derivatives were conducted. Spectrophotometric measurements of absorbance during 10 min did not reveal any interactions between the reagents.

The activity of both cholinesterases (AChE and BuChE) were conducted using a spectrophotometric method developed by Ellman with previously described modifications^25^^,^^30^. The experiments were performed in Semi-Micro cuvettes (Medlab Products, Raszyn, Poland), by means of a Cecil CE 2021 spectrophotometer (CECIL Instruments Limited, Cambridge, UK) with circulating thermostated water (37 °C) and a magnetic stirrer (Electronic Stirrer Model 300; Rank Brothers Ltd, Cambridge, England). 400-fold diluted solution of haemolysed erythrocytes or diluted plasma (200 times) was incubated for 15 min (37° C) with DTNB and tested compound at an appropriate concentration, and the reaction was started by addition of substrate (ATC or BTC). The final volume of a sample was 500 µL. The absorbance was measured at *λ* = 436 nm for three minutes continuously, and the maximal velocity of the reaction was counted on the basis of changes in absorbance over time.

The method was validated, eight control tests were conducted both for AChE and BuChE experiments. The coefficients of variability were counted (*W*_AChE_ = 0.076, *W*_BuChE_ = 0.099, respectively).

### Kinetic parameters estimation

The experiments were carried out using decreasing concentrations (2-, 3-, 5-, 10-, 20-fold) of the substrate (ATC, BTC) and two concentrations of inhibitors: one equal to its IC_50_ and 1/3 of IC_50_ value. The absorbance was recorded at *λ* = 436 nm using a CECIL 2021 spectrophotometer (CECIL Instruments Limited, Cambridge, UK) with a thermostatic water flow (temperature 37° C).

Due to the high variations in the individual concentration and activity of ChE in human erythrocytes and plasma the studies of kinetic parameters of 32 biological samples were conducted. Kinetic parameters for AChE (mean ± SD; *n* = 32): *K*_m_ = 91.97 ± 23.37 µmol/L, *V*_max_ = 0.241 ± 0.040 A/min. Kinetic parameters calculated for BuChE (mean ± SD; *n* = 32): *K*_m_ = 69.57 ± 21.34 µmol/L, *V*_max_ = 0.235 ± 0.027 A/min.

### Inhibition of cholinesterases by binary mixtures of donepezil and biguanides

To determine the potential synergistic effects of metformin derivatives on ChE inhibition binary-mixtures trials were performed. The effects of mixtures of metformin derivatives and donepezil on both ChEs activity were determined using a modified method of Ellman^25^. In brief, the samples (470 µL) were preincubated with a mixture (20 µL) of donepezil and biguanide (metformin, phenformin, or compound **5**), before substrate addition (ATC or BTC at the final concentration of 0.75 µmol/mL) for 15 min. The concentration of donepezil was between 0.01 and 100 nmol/L for AChE inhibition, and 0.2 to 100 µmol/L for BuChE measurements. In turn, the concentration of metformin, phenformin, and compound **5** were constant in every measurement, and were chosen on the basis of the respective IC_50_ values and percentage of ChE inhibition. To further characterise AChE inhibitory mode of binary mixtures kinetic evaluation was performed. Tested compounds were added into the assay solution and pre-incubated with the enzyme at 37 °C for 15 min, followed by addition of ATC at decreasing concentrations (2-, 3-, 5-, 10-, 20-fold). The characterisation of the ATC hydrolysis was conducted at 436 nm for 3 min.

### *In vitro* assays with metformin, compound 5, and excess of substrate

To assay the *in vitro* effects of metformin and compound **5** on ChE activity, the biological samples were incubated with various concentrations of metformin or derivative **5** for 15 min at 37 °C before appropriate substrate addition. The activity was measured with BTC at concentrations of 2.5 μmol/mL. To verify how BTC concentration affects anti-BuChE activities of metformin and compound **5** additional studies with the BTC ranging from 0.0375 to 2.5 μmol/mL were performed.

### Beta-amyloid aggregation studies

Beta-amyloid (Aβ 1–42) aggregation studies were conducted using SensoLyte ThT Aβ42 Aggregation kit (AnaSpec, Inc, Fremont, CA). Aβ42 peptide was dissolved in cold (4 °C) assay buffer and left to hydrate for a few minutes. The solution was spined at 10,000 rpm for 5 min at 4 °C to centrifuge out any precipitated material. The fibrillation reaction was set up by addition of thioflavin (ThT) dye at concentration of 2 mmol/L. Test samples (metformin and compound **5**) were added in a volume of 5 µL and the reaction was started by addition of Aβ42 peptide solution. The final volume of the test samples was 100 µL. Simultaneously, the positive control and negative control containing morin at the final concentration of 100 µmol/L were established. The fluorescence intensity was measured at 37 °C with Ex/Em = 440 nm/484 nm and 15 s shaking between the reads using microplate reader (Synergy H1; BioTek, ‎Winooski, VT). The measurements of fluorescence intensity expressed as relative fluorescence units (RFU) were taken for 90 min with 5 min intervals. The fluorescence intensity measured for positive control equals 100% of Aβ aggregation and was used for estimation of inhibition properties of tested biguanides.

### Data analysis

The values presented in tables and figures are expressed as the mean ± standard deviation (SD). All experiments (in duplicates) were conducted on three different biological materials (haemolysed RBCs or plasma).

The IC_50_ value, defined as the drug concentration that inhibits 50% of the activity of an enzyme, was calculated using linear regression (*y* = *a* × *x* + *b*). AChE Selectivity Index (SI) was calculated with the aid of the following formula: SI = IC_50_ of BChE/IC_50_ of AChE. In turn BuChE SI was defined as IC_50_ of AChE/IC_50_ of BChE. Linear regression (Hanes–Woolf plots) were used to estimate the maximal velocity (V_max_) and the Michaelis constant (*K*_m_).

The multiple drug effects on ChEs were examined according to the median-effect principle described by Chou et al.^31^. All the calculations were performed using ComboSyn software (http://www.combosyn.com/). The method involves the plotting of dose effective curves for every single drug and their binary mixtures in a different dose. Based on the algorithms, computer software has been developed to allow automated simulation of synergism and antagonism at all dose or effect levels. The software enables to display the dose–effect curve, median-effect plot, combination index (CI) plot, isobologram, and dose-reduction index (DRI) plot. CI-isobologram equation allows quantitative determination of drug interactions, where CI <1, = 1, and >1 indicate synergism, additive effect, and antagonism, respectively^32^.

Statistical analysis of data obtained in Aβ aggregation assay was conducted with a commercially available package (GraphPad Prism 5, La Jolla, CA). The results were expressed as the mean ± standard deviation (SD) of measurements conducted in triplicates or quadruplicates. One or two-way ANOVA and subsequent *post hoc* tests were used for intergroup comparisons. The results were considered significant at *p* values lower than 0.05.

## Results

### General cholinesterase activity

**Figure 2.**
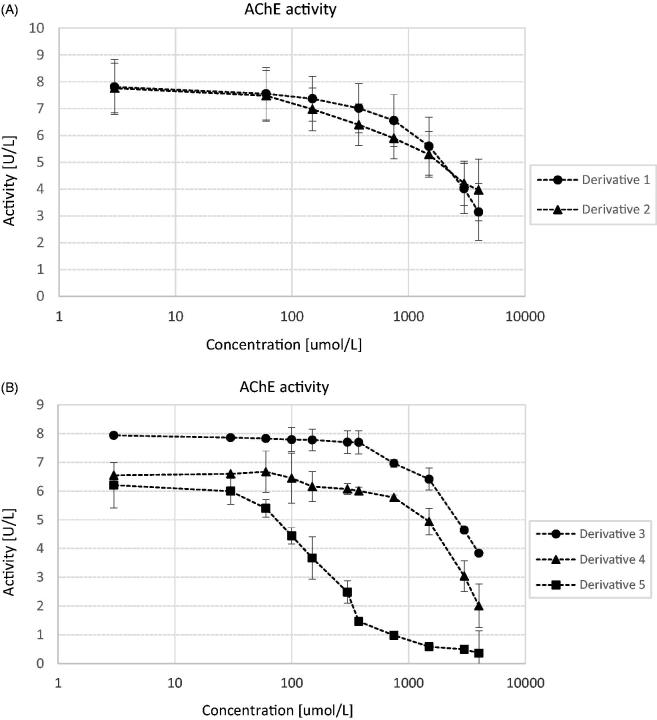
The effects of derivatives 1 and 2 (A) and 3, 4, and 5 (B) on AChE activity. The activity of the control samples was 7.51 ± 0.60 U/L. Each data point represents mean ± SD for at least three independent experiments conducted in duplicates. Transformation of these data into a percentage of enzyme inhibition, and subsequent calculations using quadratic and logarithmic equations from each conducted experiment allowed to determine the IC_50_ value for every compound.

**Figure 3.**
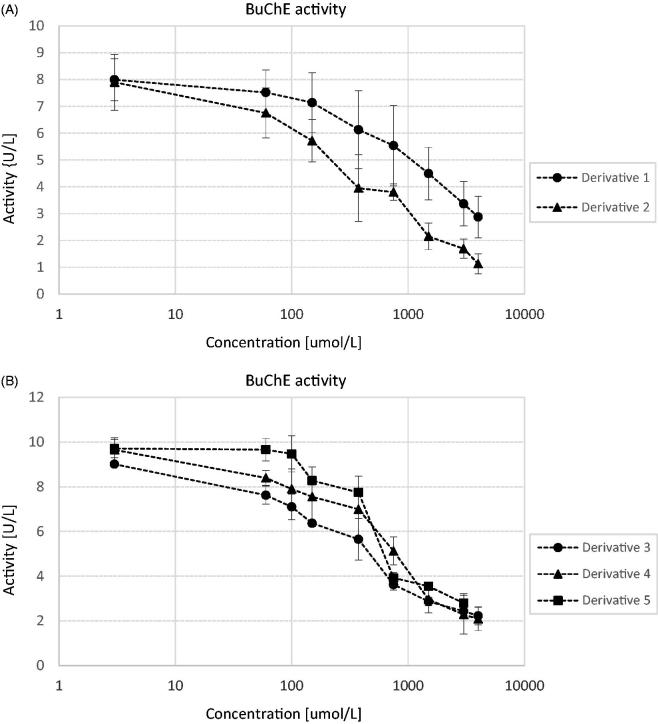
The effects of derivatives 1 and 2 (A) and 3, 4, and 5 (B) on BuChE activity. The activity of the control samples was 8.92 ± 0.84 U/L. Each data point represents mean ± SD for at least three independent experiments conducted in duplicates. Transformation of these data into a percentage of enzyme inhibition, and subsequent calculations using quadratic and logarithmic equations from each conducted experiment allowed to determine the IC_50_ value for every compound.

Within this study, the influence of two sulphenamides and three sulphonamides on the activity of human ChEs was assessed. According to the obtained results (Figure 2(A,B)) all examined compounds inhibited the activity of AChE. Similarly, in the case of BuChE (Figure 3(A,B)) all compounds possess anti-BuChE activity. On the basis of the obtained reaction velocities, the percentages of AChE and BuChE inhibition and corresponding IC_50_ values were calculated (Table 1).

**Table 1.** Effects of sulphenamide and sulphonamide derivatives of metformin on the human erythrocyte acetylcholinesterase (AChE) and plasma butyrylcholinesterase (BuChE) activity.

Compound	IC_50_ (µmol/L)		SI	
	AChE	BuChE	AChE	BuChE
1	3166.0 ± 411.2	1876.2 ± 558.3	0.59	1.69
2	3675.7 ± 692.8	334.5 ± 107.2	0.09	10.99
3	3824.7 ± 277.6	561.1 ± 191.9	0.15	6.82
4	2861.0 ± 283.0	871.3 ± 226.6	0.30	3.28
5	212.5 ± 48.3	685.8 ± 86.4	3.23	0.31
Donepezil	0.025 ± 0.004	12.8 ± 1.52	512.0	0.002
Metformin^a^	2350.0 ± 122.0	>1000.000^˟^	>425.53^˟^	<0.002^˟^
Phenformin^a^	4940.0 ± 575.0	259.0 ± 31.0	0.052	19.073

The values are given as mean ± standard deviation (SD) in three independent experiments on various biological samples. SI (Selectivity Index) – the AChE selectivity index is defined as IC_50_ BChE/IC_50_ AChE affinity ratio. Selectivity for BChE is defined as IC_50_(AChE)/IC_50_(BChE).

^a^Values of IC_50_ for metformin and phenformin were published previously^21^.*theoretical values counted on the basis of extrapolated plots for metformin towards BuChE.

Donepezil, the approved drug for the treatment of AD reversibly inactivating the ChEs, and metformin were used to compare the obtained results^4^^,^^25^. Regarding AChE inhibition derivative **5** presented the highest activity of the tested compounds (IC_50_ = 212.5 ± 48.3 µmol/L); however, this activity is much lower than that of donepezil (0.025 ± 0.004 µmol/L). All other sulphenamides and sulphonamides presented anti-AChE properties in milimolar range (Table 1). Prodrug **2** with branched alkyl chain was the most active towards inhibition of human BuChE (IC_50_ = 334.5 ± 107.2 µmol/L). Slightly higher IC_50_ values were reported for three tested sulphonamides. Calculation of SI enabled to conclude that all examined compounds apart from derivative **5** exhibited higher selectivity towards BuChE than AChE.

### Kinetic parameters

**Figure 4.**
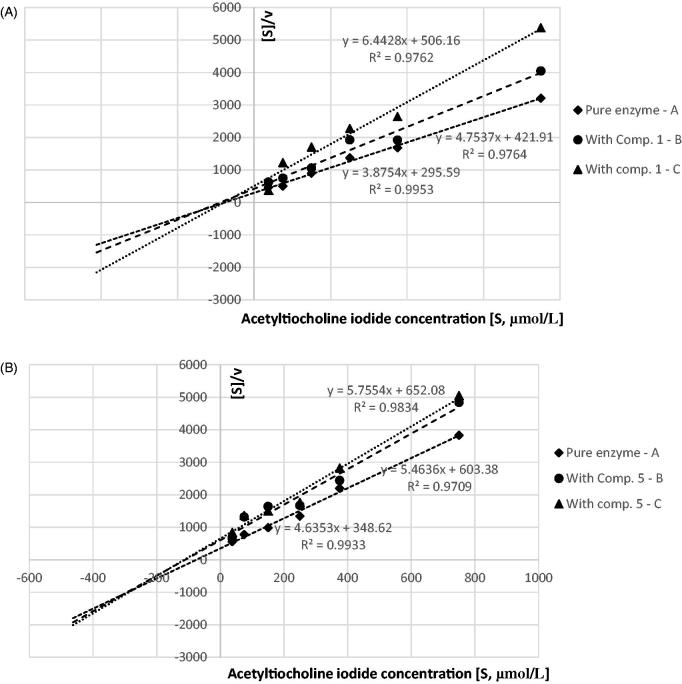
Determination of kinetic parameters of AChE enzymatic reactions. Hanes–Woolf plots we used to calculate the Michaelis constant (*K*_m_) and maximal velocity (*V*_max_). (A) AChE and compound **1** at concentration of 1055.0 µmol/L and 3166.0 µmol/L; non-competitive inhibition. (B) AChE and compound **5** at a concentration of 71.0 µmol/L and 212.0 µmol/L; mixed inhibition. Presented data constitute the results of one exemplary experiment conducted in duplicates. The results of kinetic studies conducted in three independent experiments and calculated kinetic parameters are enclosed in Table 2.

**Figure 5.**
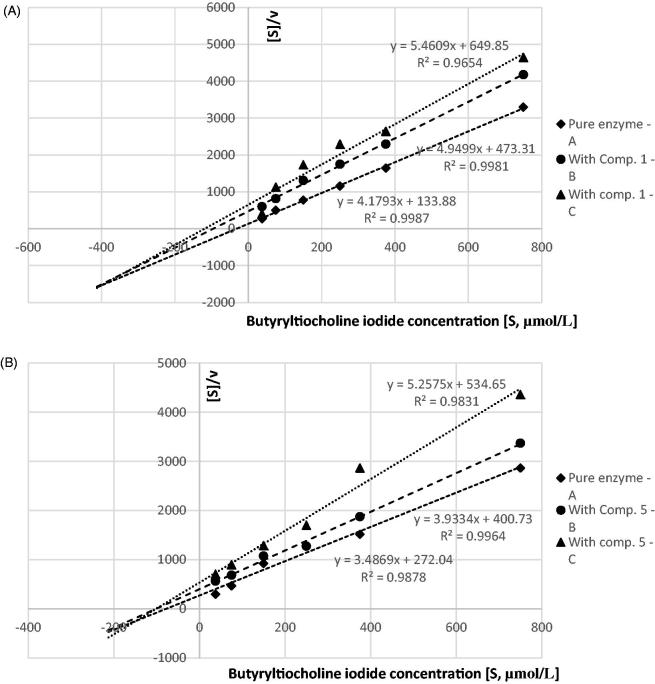
Determination of kinetic parameters of BuChE enzymatic reactions. Hanes–Woolf plots we used to calculate the Michaelis constant (*K*_m_) and maximal velocity (*V*_max_). (A) BuChE and compound **1** at a concentration of 625.0 µmol/L and 1876.0 µmol/L; mixed-type inhibition. (B) BuChE and compound **5** at a concentration of 228.0 µmol/L and 686.0 µmol/L; mixed inhibition. Presented data constitute the results of one exemplary experiment conducted in duplicates. The results of kinetic studies conducted in three independent experiments and calculated kinetic parameters are enclosed in Table 2.

To establish the type of inhibition, additional experiments were conducted with various concentrations of substrates (ATC, BTC), and the kinetic parameters of the enzymatic reactions were obtained using Hanes–Woolf equation, which is a graphical representation of enzyme kinetics in which the ratio of the initial substrate concentration [*S*] to the reaction velocity *v* is plotted against [*S*]. Hanes–Woolf (half-reciprocal) plot of [*S*]_0_/*v* against [*S*]_0_ gives intercepts at *K*_m_/*V*_max_ and *K*_m_ (Figures 4 and 5).

The kinetic parameters were estimated on the basis of three individual experiments conducted on three different biological materials. The summarised results of *K*_m_ and *V*_max_ are presented in Table 2, whereas in supplementary materials (Tables S1 and S2) detailed data on each individual reaction are included.

**Table 2.** Kinetic parameters of enzymatic reactions.

Compound		AChE			BuChE		
		*K*_m_ (µmol/L)	*V*_max_ (A/min)	I	*K*_m_ (µmol/L)	*V*_max_ (A/min)	I
**1**	A	84.81 ± 10.16	0.257 ± 0.003	NC	48.5 ± 12.0	0.240 ± 0.008	M
	B	98.15 ± 8.84	0.202 ± 0.007		109.3 ± 13.3	0.207 ± 0.015	
	C	100.45 ± 14.61	0.161 ± 0.024		198.6 ± 87.1	0.174 ± 0.012	
**2**	A	115.38 ± 10.6	0.267 ± 0.016	M	73.0 ± 8.4	0.233 ± 0.006	M
	B	129.2 ± 9.7	0.218 ± 0.005		118.6 ± 12.6	0.182 ± 0.009	
	C	137.67 ± 10.3	0.160 ± 0.011		171.9 ± 80.6	0.103 ± 0.019	
**3**	A	116.6 ± 48.1	0.195 ± 0.043	M	80.5 ± 11.7	0.265 ± 0.012	M
	B	201.7 ± 73.4	0.147 ± 0.022		88.2 ± 24.1	0.208 ± 0.005	
	C	271.6 ± 49.9	0.112 ± 0.024		105.6 ± 10.4	0.180 ± 0.019	
**4**	A	78.1 ± 12.1	0.193 ± 0.028	M	64.77 ± 14.37	0.240 ± 0.036	NC
	B	147.8 ± 8.9	0.163 ± 0.005		69.78 ± 17.32	0.229 ± 0.019	
	C	272.2 ± 21.2	0.119 ± 0.019		75.26 ± 13.97	0.174 ± 0.006	
**5**	A	71.27 ± 6.96	0.211 ± 0.001	M	90.16 ± 6.13	0.276 ± 0.022	M
	B	113.6 ± 6.80	0.175 ± 0.033		104.4 ± 11.30	0.242 ± 0.012	
	C	110.09 ± 4.20	0.146 ± 0.040		108.09 ± 1.39	0.202 ± 0.002	

The values are given as mean ± standard deviation (SD) in three independent experiments.

A: kinetic parameters for pure enzyme (*K*_m_, *V*_max_); B: kinetic parameters of tested compounds (inhibitors) (1/3 of IC_50_ concentrations) (*K*_m(i)_, *V*_max(i)_); C: kinetic parameters of tested compounds (inhibitors) (IC_50_ concentrations) (*K*_m(i)_, *V*_max(i)_); I: type of inhibition; M: mixed type; NC: non-competitive inhibition.

The type of inhibition was determined on the basis of *K*_m_ and *V*_max_ values of the results obtained for pure enzyme and tested compounds at two concentrations (Table 2, Figures 4(A,B) and 5(A,B)). In the case of AChE inhibition, derivatives **3, 4,** and **5** exhibited mixed inhibition, as *V*_max(i)_ (*V*_max_ of the reactions with inhibitor) significantly decreased in comparison with *V*_max_, whereas *K*_m(i)_ (*K*_m_ of the reaction with inhibitor) increased. It was also found that compound **1** inhibited AChE noncompetitively (constant *K*_m_ and *K*_m(i)_ and decreased *V*_max(i)_ value). According to the obtained data derivatives **1**, **2** and **3**, **5** inhibited BuChE in a mixed manner, whereas compound **4** was shown to inhibit BuChE non-competitively.

### Inhibition of cholinesterases by donepezil and biguanides mixtures

**Figure 6.**
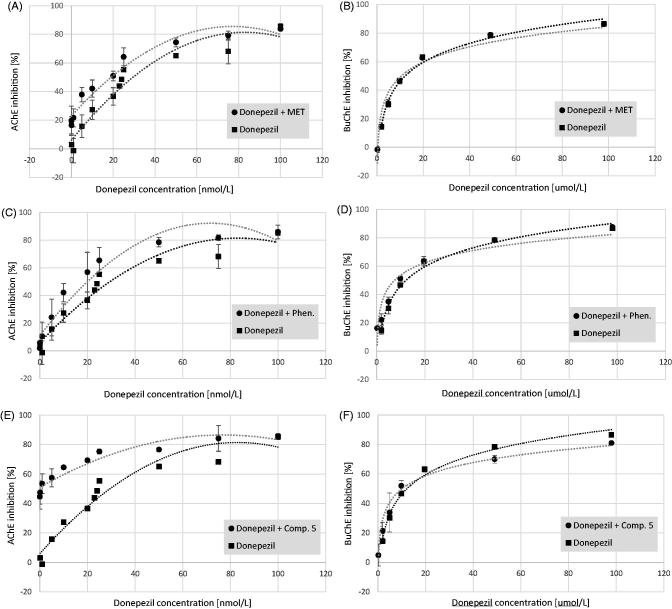
The effects of donepezil and binary mixtures of tested compounds on the activity of cholinesterases expressed as % of enzyme inhibition. (A) Acetylcholinesterase (AChE) inhibition by donepezil (0.01–100 nmol/L) and a mixture of donepezil and metformin at a concentration of 600.0 µmol/L. (B) Butyrylcholinesterase (BuChE) inhibition by donepezil (0.01–100 µmol/L) and a mixture of donepezil and metformin at a concentration of 600.0 µmol/L. (C) Acetylcholinesterase (AChE) inhibition by donepezil (0.01–100 nmol/L) and a mixture of donepezil and phenformin at a concentration of 600.0 µmol/L. (D) Butyrylcholinesterase (BuChE) inhibition by donepezil (0.01–100 µmol/L) and a mixture of donepezil and phenformin at a concentration of 150.0 µmol/L. (E) Acetylcholinesterase (AChE) inhibition by donepezil (0.01–100 nmol/L) and a mixture of donepezil and compound **5** at a concentration of 150.0 µmol/L. (F) Butyrylcholinesterase (BuChE) inhibition by donepezil (0.01–100 µmol/L) and a mixture of donepezil and compound **5** at a concentration of 300.0 µmol/L. Each data point shows the mean ± SD for three independent experiments conducted in duplicates. Dotted trend lines represent the average of three independent experiments. Quadratic and logarithmic equations from each conducted experiment allowed to determine the IC_50_ value for the mixtures.

**Figure 7.**
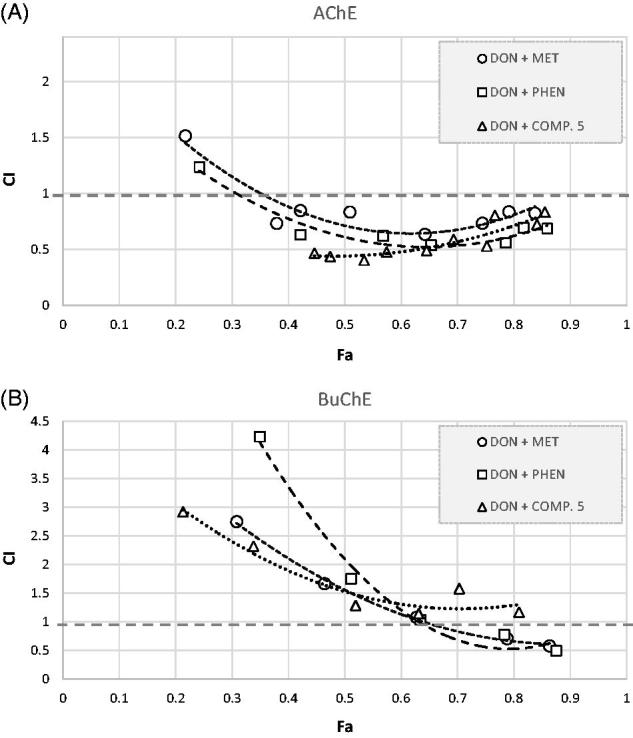
Analysis of potential synergism between donepezil and biguanides: metformin, phenformin, and compound **5** by the median effect principle. Data from the AChE (A) and BuChE (B) inhibitory activities assay were analysed by the Chou–Talalay method. The results are presented in a form of Fa-CI plots (Fa: Fraction affected; CI: Combination Index). The analysis proved that in the case of anti-AChE activity the lines of all examined binary mixtures fell in the section of synergism (CI < 1). The results regarding BuChE appear to be complex.

The presence of metformin, phenformin^25^, and compound **5** was observed to produce a concentration-dependent inhibition of AChE activity. To study the potential synergism of these compounds with donepezil several tests were conducted using binary mixtures (Figure 6(A–F)). The greatest effect towards AChE activity was found for the combination of donepezil at concentrations of 0.01–100 nmol/L and compound **5** at a constant concentration of 150.0 µmol/L corresponding to 35.03 ± 7.18% of AChE inhibition. As presented in Table 3 the IC_50_ value of donepezil/compound **5** mixture was ∼170-fold lower than IC_50_ of pure donepezil. In the case of metformin and phenformin used at a concentration of 600 µmol/L IC_50_ value decreased 1.32-, and 1.67-fold. These results were confirmed using the median-effect principle (Figure 7). Figure 7(A) shows that the potency of all three combinations on AChE activity increased compared with that of a single drug alone. In Figure 7 anti-AChE activity of binary mixtures is located in synergism section of Fa-CI graph (CI < 1.0). In addition, the drug reducing index (DRI) values of donepezil were greater than 1 which means that the dose of this selective AChE inhibitor might be 1.35- or 2.69-fold reduced when used in combination with metformin or compound **5** to obtain 50% of anti-AChE activity.

**Table 3.** Binary mixtures of metformin, phenformin, compound **5,** and donepezil and their effects on the human erythrocyte acetylcholinesterase (AChE) and plasma butyrylcholinesterase (BuChE) activity expressed as IC_50_ values.

Compound	IC_50_ (µmol/L)	
	AChE	BuChE
Donepezil + Metformin^a^	0.019 ± 0.004	10.50 ± 0.80
Donepezil + Phenformin^b^	0.015 ± 0.007	7.76 ± 1.05
Donepezil + Compound 5^c^	0.0001 ± 0.0001	10.32 ± 3.37
Donepezil	0.025 ± 0.004	12.81 ± 1.52

The results are presented as mean ± SD of three independent experiments conducted in triplicates.

Concentrations of biguanides: ^a^Metformin at 600 µmol/L (AChE and BuChE studies); ^b^Phenformin at 600 µmol/L (AChE) and 150 µmol/L (BuChE); ^c^Compound **5** at 150 µmol/L (AChE) and 300 µmol/L (BuChE).

Regarding BuChE activity the highest inhibition of this enzyme was reported for the mixture of donepezil and phenformin at a concentration of 150 µmol/L (44.73 ± 1.23% of BuChE inhibition). The IC_50_ value of this mixture was reduced by 39.47% in comparison to donepezil alone. Both metformin and compound **5** contributed to ∼20% reduction in IC_50_ values, however, these results were not confirmed by Fa-CI plots as only for the highest Fa points CI values were below 1.0 (Figure 7(B)).

To further investigate AChE inhibitory mode of metformin/compound **5** and donepezil mixtures, various doses of metformin or compound **5**, donepezil and their combinations were added to the AChE solutions containing a range of ATC (0.0375–0.75 µmol/mL). The calculated kinetic parameters of enzymatic reactions are presented in Table 4.

**Table 4.** Kinetic parameters of AChE enzymatic reactions with binary mixtures.

Binary mixture	Compound	Kinetic parameters	
		*K*_m_ (µmol/L)	*V*_max_ (A/min)]
Donepezil + Metformin	Pure AChE	61.0 ± 23.3	0.227 ± 0.011
	Metformin	111.2 ± 24.2	0.179 ± 0.020
	Donepezil	107.8 ± 25.0	0.124 ± 0.018
	Donepezil + Metformin	207.6 ± 18.7	0.127 ± 0.011
Donepezil + Compound 5	Pure AChE	68.6 ± 6.9	0.227 ± 0.007
	Compound **5**	163.9 ± 36.7	0.206 ± 0.008
	Donepezil	134.6 ± 3.4	0.145 ± 0.014
	Donepezil + Compound **5**	199.7 ± 39.0	0.134 ± 0.024

Concentrations of examined compounds: metformin at 1175 µmol/L (1/2 IC_50_ for AChE); donepezil – 0.0125 µmol/L (1/2 IC_50_ for AChE); compound **5** at 106.0 µmol/L (1/2 IC_50_ for AChE). In the experiments with binary mixtures metformin + donepezil, and compound **5** + donepezil were used also in their ½ of IC_50_ for AChE. The results are presented as mean ± SD of three independent experiments conducted in duplicates.

The data showed in Table 4 (*K*_m_ and *V*_max_) that tested compounds alone: metformin, compound **5** and donepezil inhibited AChE in a mixed-type manner as *K*_m_ values were higher in comparison with experiments without any inhibitor and simultaneously *V*_max_ values of the reactions were decreased. The results show that both tested combinations inhibited AChE activity in the same mixed type, as the interception of lines in Hanes–Wolf plots occurred in the third quadrant of the coordinate system.

### *In vitro* assays with metformin, compound 5, and excess of substrate

**Figure 8.**
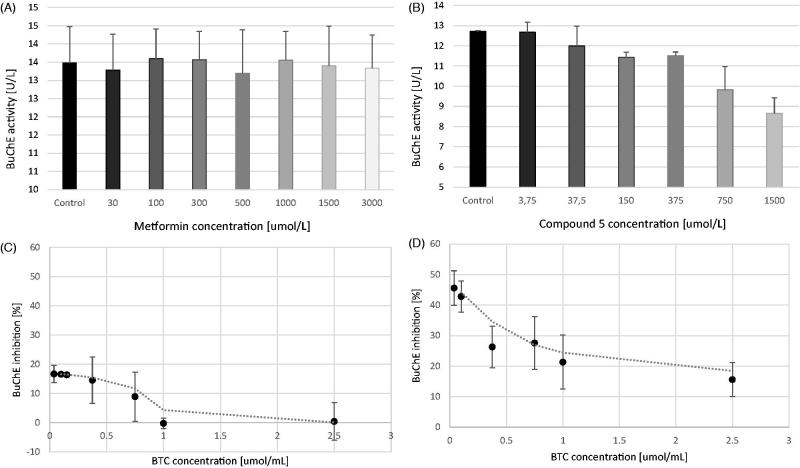
The effects of metformin (A) and compound **5** (B) on BuChE activity at BTC concentration of 2.5 μmol/mL. Metformin did not reveal anti-BuChE activity at 2.5 μmol/mL of BTC, whereas compound **5** exhibited BuChE up to 31.89 ± 6.22%. (C) It shows the dependence of BTC concentration on the anti-BuChE properties of metformin used at 1000 μmol/L. At lower concentration of BTC (up to 0.75 μmol/mL) metformin inhibited BuChE. At higher BTC concentration no anti-BuChE effects of metformin were reported. (D) It presents the relationships between BTC concentration and % of BuChE inhibition by compound **5** at 375 μmol/L. Compound **5** at 0.75 μmol/mL of BTC inhibited BuChE by 27.53 ± 8.6%, whereas at 2.5 μmol/mL of BTC the percentage of BuChE inhibition was 15.57 ± 5.6%. All the results are presented as mean ± SD of three independent measurements conducted in duplicates or triplicates.

Experiments using different concentrations of ATC and BTC (Figure S1(A,B); Supplementary materials) confirmed previously formed statements that AChE activity is inhibited by high substrate concentrations, whereas BuChE enzymatic activity increases. As in *in vivo* conditions the average concentration of ACh within the synaptic cleft has been calculated to reach 5 mM^33^ we decided to evaluate anti-BuChE properties of metformin and compound **5** at higher BTC concentrations (2.5 mmol/L). As previously published^25^ the presence of metformin was observed to produce very weak concentration-dependent inhibition of BuChE activity (up to 21.2%) at 0.75 mmol/L, but was found not to affect the enzyme activity at elevated substrate concentration (2.5 mmol/L). The percentage of BuChE inhibition by metformin at 3 mmol/L was 1.10 ± 0.51% (Figure 8(A)). These results encouraged us to perform additional studies to evaluate the relation between the concentration of substrate and anti-BuChE properties of metformin. The results presented in Figure 8(C) clearly show that inhibition of BuChE activity by metformin depends on the concentration of BTC. In contrast to metformin, compound **5** (at 375 µmol/L) inhibited BuChE activity at BTC concentration of 2.5 mmol/L up to 15.57 ± 5.6% (Figure 8(D)), however, this value was much lower than those registered for 0.75 mmol/L of BTC (27.53 ± 8.61%). Thus, anti-BuChE properties of compound **5** depend on the concentration of substrate.

### Beta-amyloid aggregation studies

**Figure 9.**
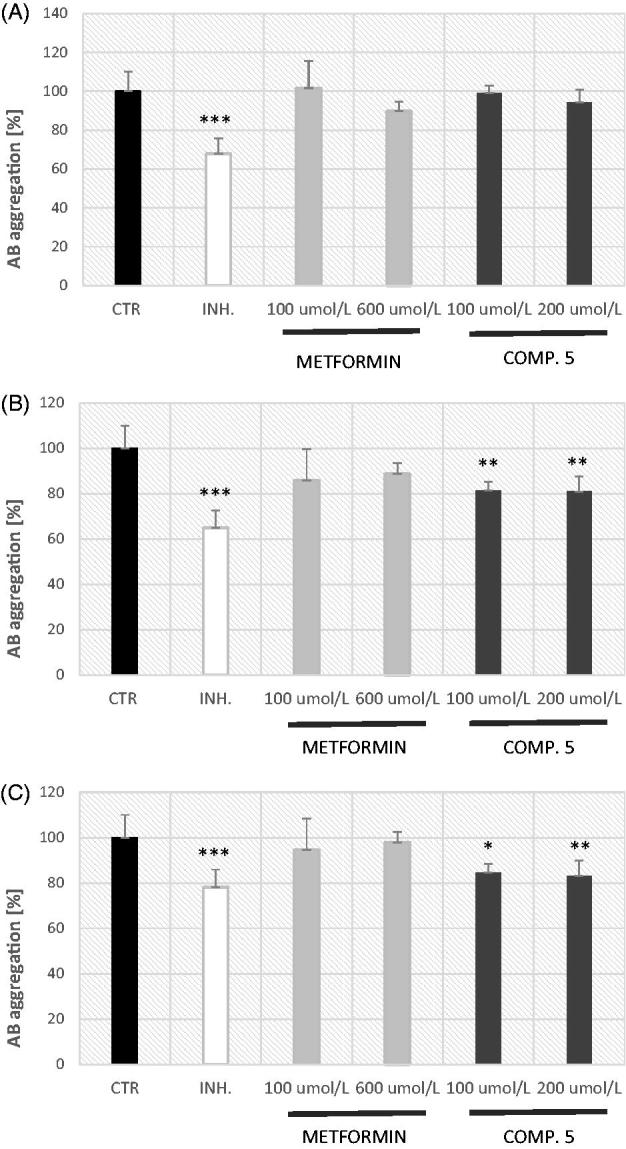
The effects of metformin and compound **5** on AB aggregation depending on the fibrillation time. (A) 30 min, (B) 60 min, and (C) 90 min. Morin at a concentration of 100 μmol/L was used as an inhibitor of AB aggregation (*p* < .001). The results are presented as mean ± SD of three or four measurements of fluorescence intensity (Ex/Em =440 nm/484 nm every 5 min at 37 °C). Metformin at both tested concentrations did not significantly affect the reaction of fibrillation over the entire measurement time in comparison with control. Compound **5** at 100 and 200 μmol/L significantly decreased AB aggregation after 60 minutes of reaction initiation. ***p < .001 vs. control, **p < .01 vs. control, *p < .05 vs. control. Two-way ANOVA analysis showed that at 90 minute of fibrillation reaction compound **5** 100 μmol/L significantly inhibited the reaction in comparison with metformin at the same concentration. No differences in inhibitory properties between inhibitor (morin) and compound **5** at 200 μmol/L at 60 and 90 min was reported.

As beta-amyloid aggregation is evidently an essential occurrence in the pathogenesis of AD, it is important to study the fibrillation reaction and to screen for Aβ aggregation inhibitors. Within this study, we performed an assay that is based on the property of ThT dye to increase its fluorescence when bound to aggregated Aβ peptides. The results of preliminary Aβ aggregation studies are shown in Figure 9(A–C). Metformin at both tested concentrations did not significantly affect the reaction of fibrillation over the entire measurement time (5–90 min) in comparison with positive control. The maximal percentage of inhibition of Aβ aggregation reported for this biguanide used at 600 µmol/L was 11.32%. Compound **5** at 100 and 200 µmol/L appeared to be a better inhibitor of a fibrillation reaction as it significantly decreased the Aβ aggregation up to ∼80% after 60 min of reaction initiation. However, two-way ANOVA analysis showed that the activity of compound **5** at a concentration of 100 µmol/L was significantly lower in comparison with morin (negative control). At higher tested concentration (200 µmol/L) the inhibitory property of compound **5** was comparable with those of morin (*p* > 0.05).

## Discussion

Commercially available synthetic AChE inhibitors (AChEIs) such as donepezil, rivastigmine, and galantamine influence the dynamics of ACh by inhibiting the activity of AChE thus increasing the availability, concentration, and interaction of this neurotransmitter with cholinergic receptors. Although the clinical benefits of these drugs are commonly regarded as relatively small, the research outcomes have demonstrated substantial effects in terms of reduced caregiver burden. In addition, there is some evidence of disease-modifying properties of AChEIs^34^. Currently, ongoing studies concentrate on the development of multifunctional compounds with capabilities of ChE inhibition, prevention, or reduction of Aβ formation and aggregation, as well as anti-oxidative properties as oxidative stress may escalate the production and aggregation of Aβ^35^. Mezeiova et al.^35^ in their latest review focus on coumarin derivatives of donepezil because these types of naturally occurring and chemically developed compounds possess a broad spectrum of pharmacological activities, which might be favourable in the treatment of AD. The authors mention a few papers pointing out that coumarins are capable of AChE inhibition by binding to its peripheral anionic site (PAS) of acetylcholinesterase, which predispose them to act as potential AChEIs^35^. Despite numerous pathophysiological aspects of AD, commercial AChEIs used for symptomatic treatment of AD shed light on the importance of AChE, which still remains a highly important classic target for the development of new potential drugs^35^.

AChE (acetylcholine hydrolase, EC 3.1.1.7) is a key enzyme in the cholinergic nervous system, responsible for hydrolysis of cationic neurotransmitter ACh. Apart from ACh hydrolysis, AChE participates also in vicious cycles resulting in aggregation of Aβ and P-tau. As presented by García-Ayllón et al. several authors have suggested that P-tau can trigger an increase in AChE expression^36^. In addition, AChE might play a role in phases of cell development, such as neuronal differentiation, regulation of cell growth or cell adhesion, which occur independently of its catalytic activity^16^^,^^37^.

The principal objective of the present research was to evaluate the effects of two sulphenamide derivatives of metformin differing in the length of alkyl chain and a series of sulphonamides on the activity of human erythrocytes AChE and plasma BuChE. This paper constitutes a continuation of our previous work^25^, which in a systematic way determined the mechanism of AChE inhibition by metformin, phenformin, and sulphenamide derivatives of metformin. In the present study two sulphenamides differing in the number of carbon atoms in the alkyl chain (n-hexyl and 2-ethylhexyl) were reported to be less potent in comparison with metformin towards AChE inhibition as their IC_50_ values were higher than 3000 µmol/L. These results seem to be in accordance with our previous study^25^ in which n-butyl sulphenamide was more active towards AChE (IC_50_ = 1190.0 ± 157.0 µmol/L), whereas n-octyl sulphenamide exhibited very low anti-AChE activity (up to 20% at 3000 µmol/L). Among tested sulphonamides the most active towards AChE appeared to be compound **5** with *o*-NO_2_ group and *p*-CF_3_ substituent (IC_50_ = 212.5 ± 48.3 µmol/L).

Regarding inhibition of BuChE the most active compound was derivative **2** with 2-ethylhexyl chain (IC_50_ = 334.5 ± 107.2 µmol/L), which is in agreement with previously reported inhibitory activity towards BuChE of n-octyl derivative (IC_50_ = 184.0 ± 14.0 µmol/L)^25^. Calculation of SI enabled to draw the conclusion that this compound exhibits the highest affinity towards BuChE of all tested compounds. Compound **2** with 2-ethylhexyl chain is more than 10-fold selective towards BuChE, whereas n-hexyl sulphenamide (**1**) expressed only ca. 1.5-fold higher affinity towards plasma BuChE. However, we can conclude that long and branched alkyl chain of sulphenamide increases the BuChE inhibitory activity and selectivity. Among the tested sulphonamides, the most active derivative towards BuChE was compound **3** with the *para-*nitro group in the aromatic ring. However, the activity of all three sulphonamides was within the comparable range.

Grossberg has pointed that despite the fact that BuChE represents only 10% of total ChE activity in a healthy human brain, the importance of BuChE in cholinergic neurotransmission increases in AD^38^. The significance of the inhibition of both ChEs is proved by the clinical results of rivastigmine administration in AD patients manifested by cognitive improvement^39^. Furthermore, many recently published studies have highlighted that BuChE plays a more important role in the AD brain and selective inhibitors of BuChE could be promising drug candidates^40^^,^^41^. According to Sridhar et al.^42^ BuChE selectivity seems to be crucial not only in AD but also with relation to inflammation, oxidative stress, and lipid metabolism. Abbott et al.^43^ have also indicated a correlation between BuChE and insulin sensitivity, which suggests that BuChE could have a crucial role in diabetes associated with insulin resistance^43^^,^^44^. Furthermore, to indicate a potential multidirectional function of BuChE, connections between its activity and lipid levels, stroke, preeclampsia, systemic lupus erythematosus, and cardiovascular disease might be mentioned^45^.

When comparing IC_50_ values of biguanides with clinically approved drug donepezil it is clear that tested compounds present significantly lower activity towards both ChEs. However, it should be stressed out that the obtained results of weak ChEs inhibition are covered within the therapeutic concentrations. Furthermore, compounds of natural origin with potential application as anti-AD drug candidates are also much less potent^30^^,^^46^.

The crystallographic structure of AChE reveals that it includes two separate ligand binding sites; a PAS at the entrance consisting of Trp86, Tyr337, Trp286, and Tyr72, and a catalytic active site (CAS) at the bottom. An active site of AChE contains 1) an esteratic site (ES) with the catalytic triad Ser200-His440-Glu327; 2) an oxyanion hole (OAH); 3) an acyl binding site (ABS); and 4) an anionic substrate binding site (AS)^16^^,^^34^. Therefore, inhibitors binding to either site CAS or PAS could inhibit AChE^47^. Donepezil inhibits AChE through binding with the active site by interactions with benzyl substituent (CAS of AChE), the atom of the piperidine (mid-gorge) and dimethoxyindanone moiety (PAS of AChE)^47^. It has been recently stated that AChE promotes amyloid fibril formation by interaction through the PAS of AchE, therefore, the development of novel agents capable of dual binding (both CAS and PAS) is a very desirable and promising approach.

Regarding the non-competitive inhibition by which sulphenamide **1** interacts with AChE (Table 2) it can be predicted that this compound binds to PAS not CAS, similarly as has been reported also for other sulphenamides^25^. However, branched sulphenamide **2** and all new sulphonamides **3**–**5** inhibited AchE with a mixed type manner, indicating that aromaticity or bulkier structure may allow biguanide derivatives also to bind to the CAS. Binding with PAS results in the changes of the enzyme’s three dimensional structure so that acetyltiocholine (ATC) still can bind to CAS with normal affinity, but it is not the optimal configuration to stabilise the transition state and catalyse the reaction. However, the largest sulphonamide **5** with *ortho*-nitro and *para*-trifluoromethyl substituents in the aromatic ring had also the lowest IC_50_ value, which may imply that the most efficient inhibition may be achieved only if both CAS and PAS are reached. Both sulphenamides (**1** and **2**) inhibited BuChE with mixed-type manner, which is consistent with our previous research^25^ and also other studies claiming that PAS is smaller in BuChE than in AChE^48^. Sulphonamides (**3** and **5**) with ortho- and *para-*nitro substituents in aromatic ring inhibit AChE in a mixed way, similarly to donepezil.

Mixed type of BuChE inhibition by compound **5** was confirmed by additional studies with a larger amount of substrate (BTC at a concentration of 2.5 μmol/mL). The ability of compound **5** to inhibit BuChE at higher doses of BTC was decreased in comparison with general BuChE studies (BTC at 0.75 μmol/mL). Compound **5** inhibited BuChE activity at BTC concentration of 2.5 mmol/L up to 15.57 ± 5.6% (Figure 8(D)), whereas at 0.75 umol/mL of BTC the percentage of BuChE inhibition was 27.53 ± 8.6% (Figure 8(B,D)). Unlike AChE, which has been found to be inhibited by ACh at high concentrations (>1 mM), BuChE activity is stimulated under the same conditions^33^. At lower concentrations of substrate, BuChE activity is related to Michaelis–Menten kinetics and its enzymatic activity is based on the formation of an enzyme–substrate complex [ES]. At higher substrate levels (> 1 mM) BuChE presents greater activity related to the formation of a substrate-activated complex [SES]^33^. Above-mentioned results indicate that compound **5** is able to inhibit BuChE through enzyme-substrate complex [ES] and, to lesser extent the substrate activated complex [SES]. This fact is of vital importance when studying BuChE inhibitors as *in vivo* the concentration of ACh in the synaptic cleft is estimated to be about 5 mM^33^, which imply that substrate activated form of BuChE is present in the synaptic cleft. Compound **5** is also characterised by favourable intracellular uptake profile. Our studies (unpublished data) using *in vitro* cellular model revealed ∼70–80-fold higher uptake than metformin, depending on the cell line. Therefore, we presume that *para*-trifluoromethyl-*ortho*-nitro sulphonamide might be able to penetrate BBB. In addition, according to our previous results compound **5** does not contribute to the erythrocytes membrane disintegration over the entire concentration range (6–1500 μmol/L)^29^. Furthermore, our recent studies evaluating the effects of biguanide derivatives on the viability and integrity of human umbilical vein endothelial cells (HUVECs) using real-time electrical impedance system showed that compound **5** at the concentration range 6–100 μmol/L did not affect cell viability, whereas at the concentration of 300 μmol/L it contributed to ∼20% decrease in the cell adhesion and viability during 12–72 h of incubation (unpublished data). Therefore, we presume that at concentration being equal to IC_50_ value for AChE inhibition (ca. 200 μmol/L) the compound does not exert a toxic effect.

According to the clinical point of view, the need for better treatment of subjects with AD has prompted many responses, including the empirical use of drug combinations^33^. For instance, in AD patients the serotonergic system becomes also affected, which may lead to some symptoms of the disease. This observation has led Walsh et al.^33^ to conduct the studies that revealed that citalopram–galantamine combination produces the effect of BuChE inhibition considered to be synergistic. Similarly, the results of epidemiological and pathophysiological studies indicate the co-existence of type 2 diabetes mellitus (T2DM) and AD. These two conditions are characterised by similar glucose levels, insulin resistance, and biochemical factors such as inflammation and oxidative stress^33^. The diabetic state was also found to contribute to increased AChE activity, which is one of the factors leading to neurodegeneration in AD. Therefore, within this study, we decided to examine the potential synergistic effect of tested biguanides with donepezil. The experiments on the anti-AChE effects of binary mixtures consisting of donepezil and metformin, phenformin or derivative **5** exhibited the potential synergism between these compounds. Obtained results were confirmed by two independent calculations, determination of IC_50_ values of binary mixtures and combination index (CI) using the median-effect principle. A combination of donepezil and biguanides produced an anti-AChE effect, which was greater than either compound alone. This synergistic effect, which could result in higher brain ACh levels, would provide an additional rationale for any clinical benefit for AD subject suffering simultaneously from T2DM. A combination approach for dose optimisation was also used by Mak et al.^49^ who established that berberine and palmatine could produce synergistic effects to inhibit human recombinant AChE. The authors concluded that herbs containing these two alkaloids might be potentially used to prepare the herbal decoction for AD treatment^49^. The findings presented herein suggest that the interplay between anti-diabetic drug metformin, its derivatives and cholinesterase inhibitors may be complex and need to be further examined to rationalise their beneficial effects on the symptomatic treatment of AD. Taking into consideration the potentiating effects of metformin and the fact that currently available treatment for AD does not modify the course of disease we presume that the combination therapy of AD subjects could be beneficial because of its efficacy and the possibility to reduce the dose of donepezil if needed. This, in turn, could result in fewer side effects, which is frequently associated with donepezil use.

Apart from dysfunction of cholinergic neurotransmission, the pathological characteristics of AD include extracellular amyloid plaques consisting of aggregated Aβ, intracellular neurofibrillary tangles (NFTs) comprising hyperphosphorylated tau protein, and neuronal loss^50^. Aβ develops from consecutive cleavage of the amyloid β precursor protein (APP) by β-site APP cleavage enzyme 1 (BACE1) and the γ-secretase complex^51^. The review of the state-of-the-art literature on AD allows to observe that the MTDL (multi-target-directed ligand) design strategy is widely used to develop single chemical compounds that are able to simultaneously modulate multiple pathophysiological nature of AD involving cholinergic dysfunction, amyloid aggregation, and oxidative stress^52^. However, none of the disease modifying drugs that were recently developed has demonstrated sufficient efficacy in phase III studies, reducing Aβ production, preventing its aggregation or promoting Aβ clearance^52^. The reason for these poor outcomes might be large differences in effective concentration for different biological targets. For instance, a series of indanone derivatives combined with the excellent AChE inhibitory properties at the nanomolar range with anti-Aβ aggregation properties at micromolar concentration^53^. Similar conclusions were drawn in the extensive review of Mezeiova et al.^35^. The authors state that it is highly demanding task to obtain MTDLs with balanced activity/affinity profile towards different targets and scientists ought to bear in mind that compounds need to be optimised by addressing activities in the same concentration ranges^35^. Therefore, there is a great need to search for new drug candidates that are able to modulate multiple targets simultaneously with comparable affinities. Our results of Aβ aggregation studies showed that compound **5** with CF_3_ and NO_2_ substituents at 100 and 200 µmol/L presented moderate, yet significant anti-Aβ aggregation properties (c.a. 20% of fibrillation inhibition). Taking into consideration MTDLs design strategy it should be highlighted that this compound presents anti-AChE properties within the same range (IC_50_ = 212.5 ± 48.3 µmol/L).

Metformin was found not to significantly affect the process of Aβ aggregation *in vitro*. This observation is of vital importance in the view of the ambiguous results in previously published papers. For example, Hettich et al.^54^ stated that metformin markedly deceased BACE1 protein expression and activity in cell culture models, thereby reducing the BACE1 cleavage products and the production of Aβ^54^. Furthermore, Li et al.^55^ reported that high levels of Aβ1–42 in the hippocampi of mice were attenuated by metformin. In contrast, metformin was also found to increase the generation of Aβ protein^56^, which, in turn, indicates that long-term therapy with metformin may be associated with a slightly higher risk of the development of AD^57^.

## Conclusions

This work is a continuation of our previous studies^25^ whose aim was to determine the inhibitory properties of metformin and its derivatives towards human ChEs. Within this paper two sulphenamides differing in the length of alkyl chain and three sulphonamides were examined for their anti-cholinesterase properties. Derivative **5** with CF_3_ and NO_2_ in the aromatic ring was found to be the most active towards AChE (IC_50_ = 212.5 ± 48.3 µmol/L); however, this activity is much lower than that of donepezil. The other compounds were less active towards AChE in comparison with metformin for which moderate AChE inhibitory properties were confirmed^25^. In turn, derivative **2** with branched alkyl chain was shown to possess the highest anti-BuChE properties (IC_50_ = 334.5 ± 107.2 µmol/L), which seems to confirm our previous statement that the bulky side chains of sulphenamides are most likely to interact with the PAS of AChE and predispose the compounds towards BuChE-selective inhibition. The studies on the potential synergism between biguanides and donepezil showed that a combination of donepezil and metformin or compound **5** produces an anti-AChE effect, which is greater than either compound alone. Reported synergistic effect between donepezil and metformin, which could result in higher brain ACh levels, might have potential in preventing brain disorders associated with diabetes complications in future or might provide an additional rationale for the clinical benefit for AD subject suffering simultaneously from T2DM. Furthermore, it was found that compound **5** at 200 µmol/L significantly inhibits Aβ aggregation by ∼20% in comparison to control. Taken together, the results presented within this paper may contribute to a better understanding of how the action of clinically approved AChE inhibitors may be enhanced by co-treatment with other commonly used medications including metformin in everyday practice. Given the promising synergistic effects results between donepezil and biguanides, we believe that metformin may be regarded as an effective adjuvant to donepezil. Furthermore, derivatives with biguanide scaffold might be considered as a promising starting point for anti-AD drug design in the future.

## Supplementary Material

Supplemental Material

## References

[1] Sahoo AK, Dandapat J, Dash UC, et al. Features and outcomes of drugs for combination therapy as multi-targets strategy to combat Alzheimer’s disease. J Ethnopharmacol 2018;215:42–73. 29248451 10.1016/j.jep.2017.12.015

[2] Amatsubo T, Yanagisawa D, Morikawa S, et al. Amyloid imaging using high-field magnetic resonance. Magn Reson Med Sci 2010;9:95–9. 20885081 10.2463/mrms.9.95

[3] Hersi M, Irvine B, Gupta P, et al. Risk factors associated with the onset and progression of Alzheimer’s disease: a systematic review of the evidence. Neurotoxicology 2017;61:143–87. 28363508 10.1016/j.neuro.2017.03.006

[4] Zhang Y, Huang NQ, Yan F, et al. Diabetes mellitus and Alzheimer’s disease: GSK-3β as a potential link. Behav Brain Res 2018;339:57–65. 29158110 10.1016/j.bbr.2017.11.015

[5] Kumar D, Ganeshpurkar A, Kumar D, et al. Secretase inhibitors for the treatment of Alzheimer’s disease: long road ahead. Eur J Med Chem 2018;148:436–52. 29477076 10.1016/j.ejmech.2018.02.035

[6] Kuca K, Soukup O, Maresova P, et al. Current approaches against Alzheimer’s disease in clinical trials. J Braz Chem Soc 2016;27:641–9.

[7] Kumar K, Kumar A, Keegan RM, et al. Recent advances in the neurobiology and neuropharmacology of Alzheimer’s disease. Biomed Pharmacother 2018;98:297–307. 29274586 10.1016/j.biopha.2017.12.053

[8] Tonelli DM, Catto DM, Tasso DB, et al. Multitarget therapeutic leads for Alzheimer’s disease. Quinolizidinyl derivatives of bi- and tri-cyclic systems as dual inhibitors of cholinesterases and Aβ aggregation. ChemMedChem 2015;10:1040–53. 25924599 10.1002/cmdc.201500104

[9] Folch J, Ettcheto M, Petrov D, et al. Review of the advances in treatment for Alzheimer disease: strategies for combating β-amyloid protein. Neurol 2017;33:47–58. 10.1016/j.nrl.2015.03.012 25976937

[10] Korábečný J, Nepovimová E, Cikánková T, et al. Newly developed drugs for Alzheimer’s disease in relation to energy metabolism, cholinergic and monoaminergic neurotransmission. Neuroscience 2018;370:191–206. 28673719 10.1016/j.neuroscience.2017.06.034

[11] Cheignon C, Tomas M, Bonnefont-Rousselot D, et al. Oxidative stress and the amyloid beta peptide in Alzheimer’s disease. Redox Biol 2018;14:450–64. 29080524 10.1016/j.redox.2017.10.014 PMC5680523

[12] Villa V, Thellung S, Bajetto A, et al. Novel celecoxib analogues inhibit glial production of prostaglandin E2, nitric oxide, and oxygen radicals reverting the neuroinflammatory responses induced by misfolded prion protein fragment 90-231 or lipopolysaccharide. Pharmacol Res 2016;113:500–14. 27667770 10.1016/j.phrs.2016.09.010

[13] Villa V, Thellung S, Corsaro A, et al. Celecoxib inhibits prion protein 90-231-mediated Pro-inflammatory responses in microglial cells. Mol Neurobiol 2016;53:57–72. 25404089 10.1007/s12035-014-8982-4

[14] Markowicz-Piasecka M, Huttunen KM, Mateusiak Ł, et al. Is metformin a perfect drug? Updates in pharmacokinetics and pharmacodynamics. Curr Pharm Des 2017;23:2532–50. 27908266 10.2174/1381612822666161201152941

[15] Kalyanaraman B, Cheng G, Hardy M, et al. Modified metformin as a more potent anticancer drug: mitochondrial inhibition, redox signaling, antiproliferative effects and future EPR studies. Cell Biochem Biophys 2017;75:311–17. 28429253 10.1007/s12013-017-0796-3 PMC5680142

[16] Markowicz-Piasecka M, Sikora J, Szydłowska A, et al. Metformin – a future therapy for neurodegenerative diseases: theme: drug discovery, development and delivery in Alzheimer’s disease Guest Editor: Davide Brambilla. Pharm Res 2017;34:2614. 28589443 10.1007/s11095-017-2199-y PMC5736777

[17] Guo M, Mi J, Jiang QM, et al. Metformin may produce antidepressant effects through improvement of cognitive function among depressed patients with diabetes mellitus. Clin Exp Pharmacol Physiol 2014;41:650–6. 24862430 10.1111/1440-1681.12265

[18] Herath PM, Cherbuin N, Eramudugolla R, et al. The effect of diabetes medication on cognitive function: evidence from the PATH through life study. Biomed Res Int 2016;2016:1. 10.1155/2016/7208429 PMC4853928 27195294

[19] Scarpello JH, Howlett HC Metformin therapy and clinical uses. Diabetes Vasc Dis Res. 2008;5:157–67. 10.3132/dvdr.2008.027 18777488

[20] Azadeh E-H, Asadbegi M, Salehi I, et al. Neuroprotective role of antidiabetic drug metformin against amyloid beta peptide-induced neuronal loss in hippocampal CA1 pyramidal neurons in rats fed high fat diet. J Chem Pharm Sci 2016;9:3460–5.

[21] Patrone C, Eriksson O, Lindholm D Diabetes drugs and neurological disorders: new views and therapeutic possibilities. Lancet Diabetes Endocrinol 2014;2:256–62. 24622756 10.1016/S2213-8587(13)70125-6

[22] Wang J, Gallagher D, Devito LM, et al. Metformin activates an atypical PKC-CBP pathway to promote neurogenesis and enhance spatial memory formation. Cell Stem Cell 2012;11:23–35. 22770240 10.1016/j.stem.2012.03.016

[23] Bhutada P, Mundhada Y, Bansod K, et al. Protection of cholinergic and antioxidant system contributes to the effect of berberine ameliorating memory dysfunction in rat model of streptozotocin-induced diabetes. Behav Brain Res 2011;220:30–41. 21262264 10.1016/j.bbr.2011.01.022

[24] Saliu JA, Oboh G, Omojokun OS, et al. Effect of dietary supplementation of Padauk (*Pterocarpus soyauxii*) leaf on high fat diet/streptozotocin induced diabetes in rats’ brain and platelets. Biomed. Pharmacother 2016;84:1194–201. 27788477 10.1016/j.biopha.2016.10.043

[25] Markowicz-Piasecka M, Sikora J, Mateusiak A, et al. Metformin and its sulfenamide prodrugs inhibit human cholinesterase activity. Oxid Med Cell Longev 2017;2017:1. 10.1155/2017/7303096 PMC5523189 28770024

[26] Huttunen KM, Leppänen J, Laine K, et al. Convenient microwave-assisted synthesis of lipophilic sulfenamide prodrugs of metformin. Eur J Pharm Sci 2013;49:624–8. 23732628 10.1016/j.ejps.2013.05.023

[27] Rautio J, Vernerová M, Aufderhaar I, et al. Glutathione-S-transferase selective release of metformin from its sulfonamide prodrug. Bioorganic Med Chem Lett 2014;24:5034–6. 10.1016/j.bmcl.2014.09.019 25248681

[28] Kajbaf F, De Broe ME, Lalau J-D Therapeutic concentrations of metformin: a systematic review. Clin Pharmacokinet 2016;55:439–59. 26330026 10.1007/s40262-015-0323-x

[29] Markowicz-Piasecka M, Huttunen KM, Mikiciuk-Olasik E, Sikora J Biocompatible sulfenamide and sulfonamide derivatives of metformin can exert beneficial effects on plasma haemostasis. Chem Biol Interact 2018;280:15. 29217384 10.1016/j.cbi.2017.12.005

[30] Kuźma Ł, Wysokińska H, Sikora J, et al. Taxodione and extracts from *Salvia austriaca* roots as human cholinesterase inhibitors. Phyther Res 2016;30:234–42. 10.1002/ptr.5521 26621777

[31] Chou T-C, Motzer RJ, Tong Y, Bosl GJ Computerized quantitation of synergism and antagonism of taxol, topotecan, and cisplatin against human teratocarcinoma cell growth: a rational approach to clinical protocol design. J Natl Cancer Inst 1994;86:1517–24. 7932806 10.1093/jnci/86.20.1517

[32] Chou T-C Theoretical basis, experimental design, and computerized simulation of synergism and antagonism in drug combination studies. Pharmacol Rev 2006;58:621–81. 16968952 10.1124/pr.58.3.10

[33] Walsh R, Rockwood K, Martin E, Darvesh S Synergistic inhibition of butyrylcholinesterase by galantamine and citalopram. Biochin Biophys Acta 2011;1810:1230–5. 10.1016/j.bbagen.2011.08.010 PMC3263522 21872646

[34] Imramovsky A, Stepankova S, Vanco J, et al. Acetylcholinesterase-inhibiting activity of salicylanilide N-alkylcarbamates and their molecular docking. Molecules 2012;17:10142–58. 22922284 10.3390/molecules170910142 PMC6268027

[35] Mezeiova E, Spilovska K, Nepovimova E, et al. Profiling donepezil template into multipotent hybrids with antioxidant properties. J Enzyme Inhib Med Chem 2018;33:583–606. 29529892 10.1080/14756366.2018.1443326 PMC6009928

[36] García-Ayllón M-S Revisiting the role of acetylcholinesterase in Alzheimer’s disease: cross-talk with P-tau and β-amyloid. Front Mol Neurosci 2011;4:1–9. 21949503 10.3389/fnmol.2011.00022 PMC3171929

[37] Arendt T, Brückner MK Perisomatic sprouts immunoreactive for nerve growth factor receptor and neurofibrillary degeneration affect different neuronal populations in the basal nucleus in patients with Alzheimer’s disease. Neurosci Lett 1992;148:63–6. 1300505 10.1016/0304-3940(92)90805-h

[38] Grossberg GT Cholinesterase inhibitors for the treatment of Alzheimer's disease: getting on and staying on. Curr Ther Res Clin Exp 2003;64:216–35. 24944370 10.1016/S0011-393X(03)00059-6 PMC4052996

[39] Giacobini E, Spiegel R, Enz A, et al. Inhibition of acetyl- and butyryl-cholinesterase in the cerebrospinal fluid of patients with Alzheimer’s disease by rivastigmine: correlation with cognitive benefit. J Neural Transm (Vienna) 2002;109:1053–65. 12111443 10.1007/s007020200089

[40] Greig NH, Utsuki T, Ingram DK, et al. Selective butyrylcholinesterase inhibition elevates brain acetylcholine, augments learning and lowers Alzheimer-amyloid peptide in rodent. Proc Natl Acad Sci 2005;102:17213–8. 10.1073/pnas.0508575102 PMC1288010 16275899

[41] Nordberg A, Ballard C, Bullock R, et al. A review of butyrylcholinesterase as a therapeutic target in the treatment of Alzheimer’s disease. Prim Care Companion CNS Disord 2013;15:12r01412. 10.4088/PCC.12r01412 PMC3733526 23930233

[42] Sridhar GR, Rao AA, Srinivas K, et al. Butyrylcholinesterase in metabolic syndrome. Med Hypotheses 2010;75:648–51. 20797821 10.1016/j.mehy.2010.08.008

[43] Abbott CA, MacKness MI, Kumar S, et al. Relationship between serum butyrylcholinesterase activity, hypertriglyceridemia and insulin sensitivity in diabetes mellitus. Clin Sci 1993;85:77–81. 8149699 10.1042/cs0850077

[44] Iwasaki T, Yoneda M, Nakajima A, et al. Serum butyrylcholinesterase is strongly associated with adiposity, the serum lipid profile and insulin resistance. Intern Med 2007;46:1633–9. 17917325 10.2169/internalmedicine.46.0049

[45] Shahmohamadnejad S, Vaisi-Raygani A, Shakiba Y, et al. Association between butyrylcholinesterase activity and phenotypes, paraoxonase192 rs662 gene polymorphism and their enzymatic activity with severity of rheumatoid arthritis: correlation with systemic inflammatory markers and oxidative stress, preliminary. Clin Biochem 2015;48:63–9. 25179377 10.1016/j.clinbiochem.2014.08.016

[46] Khan H, Marya, Amin S, et al. Flavonoids as acetylcholinesterase inhibitors: current therapeutic standing and future prospects. Biomed Pharmacother 2018;101:860–70. 29635895 10.1016/j.biopha.2018.03.007

[47] Wang J, Wang ZM, Li XM, et al. Synthesis and evaluation of multi-target-directed ligands for the treatment of Alzheimer’s disease based on the fusion of donepezil and melatonin. Bioorganic Med Chem 2016;24:4324–38. 10.1016/j.bmc.2016.07.025 27460699

[48] Bajda M, Więckowska A, Hebda M, et al. Structure-based search for new inhibitors of cholinesterases. Int J Mol Sci 2013;14:5608–32. 23478436 10.3390/ijms14035608 PMC3634507

[49] Mak S, Luk WWK, Cui W, et al. Synergistic inhibition on acetylcholinesterase by the combination of berberine and palmatine originally isolated from Chinese medicinal herbs. J Mol Neurosci 2014;53:511–16. 24793543 10.1007/s12031-014-0288-5

[50] Johnson GVW Tau phosphorylation in neuronal cell function and dysfunction. J Cell Sci 2004;117:5721–9. 15537830 10.1242/jcs.01558

[51] Sun X, Bromley‐Brits K, Song W Regulation of beta-site APP cleaving enzyme 1 gene expression and its role in Alzheimer’s disease. J Neurochem 2012;120:62–70. 22122349 10.1111/j.1471-4159.2011.07515.x

[52] Agis-Torres A, Sollhuber M, Fernandez M, et al. Multi-target-directed ligands and other therapeutic strategies in the search of a real solution for Alzheimer’s disease. Curr Neuropharmacol 2014;12:2–36. 24533013 10.2174/1570159X113116660047 PMC3915347

[53] Akrami H, Mirjalili BF, Khoobi M, et al. Indolinone-based acetylcholinesterase inhibitors: synthesis, biological activity and molecular modeling. Eur J Med Chem 2014;84:375–81. 25036795 10.1016/j.ejmech.2014.01.017

[54] Hettich MM, Matthes F, Ryan DP, et al. The anti-diabetic drug metformin reduces BACE1 protein level by interfering with the MID1 complex. PLoS One 2014;9:e102420. 25025689 10.1371/journal.pone.0102420 PMC4099345

[55] Li J, Deng J, Sheng W, et al Metformin attenuates Alzheimer's disease-like neuropathology in obese, leptin-resistant mice . Pharmacol Biochem Behav 2012;101:564–74. 22425595 10.1016/j.pbb.2012.03.002 PMC3327803

[56] Chen Y, Zhou K, Wang R, et al. Antidiabetic drug metformin (GlucophageR) increases biogenesis of Alzheimer’s amyloid peptides via up-regulating BACE1 transcription. Proc Natl Acad Sci 2009;106:3907–12. 19237574 10.1073/pnas.0807991106 PMC2656178

[57] Imfeld P, Bodmer M, Jick SS, et al. Metformin, other antidiabetic drugs, and risk of Alzheimer’s disease: a population-based case-control study. J Am Geriatr Soc 2012;60:916–21. 22458300 10.1111/j.1532-5415.2012.03916.x

