# Supplementary material for: Metformin and its sulphonamide derivative simultaneously potentiateanti-cholinesterase activity of donepezil and inhibit beta-amyloid aggregation
Source: J Enzyme Inhib Med Chem. 2018 Sep 24;33(1):1309–22. doi: 10.1080/14756366.2018.1499627 (PMC6161601; doi:10.1080/14756366.2018.1499627)
Supplement: Supplemental Material [file IENZ_A_1499627_SM5455.pdf]

Table S1. *Kinetic parameters of enzymatic AChE reactions.*

COMPOUND		Km [μmol/L]	v_{max} [mA/min]	Mean ± SD		
				A. B. C	K_m [μmol/L]	v_{max} [mA/min]
**1**	A_1	76.276	258.038	A	84.81 ± 10.16	265.96 ± 6.929
	B_1	88.752	210.362			
	C_1	78.568	155.200			
	A_2	96.059	268.998	B	98.15 ± 8.84	202.18 ± 7.39
	B_2	106.306	195.955			
	C_2	127.105	188.583			
	A_3	82.099	270.863	C	100.45 ± 24.61	161.59 ± 24.42
	B_3	99.409	200.260			
	C_3	95.686	141.006			
**2**	A_1	133.248	283.930	A	115.38 ± 15.68	267.22 ± 16.12
	B_1	140.224	215.564			
	C_1	159.640	156.143			
	A_2	103.894	251.743	B	129.23 ± 9.67	218.43 ± 5.69
	B_2	125.478	214.749			
	C_2	119.386	173.124			
	A_3	109.000	266.000	C	137.67 ± 20.37	160.75 ± 10.82
	B_3	122.104	225.000			
	C_3	134.000	153.011			
**3**	A_1	75.213	178.824	A	116.63 ± 48.13	195.19 ± 42.94
	B_1	117.138	136.619			
	C_1	214.826	87.466			
	A_2	105.261	162.843	B	201.72 ± 73.39	147.43 ± 22.32
	B_2	239.374	132.580			
	C_2	291.898	113.130			
	A_3	169.443	243.908	C	271.64 ± 49.87	112.29 ± 24.418
	B_3	248.650	173.106			
	C_3	308.212	136.281			
**4**	A_1	72.163	165.057	A	78.06 ± 12.11	192.54 ± 27.98
	B_1	144.380	168.393			
	C_1	293.428	141.058			
	A_2	70.044	191.586	B	147.75 ± 8.92	162.74 ± 5.00
	B_2	157.877	158.846			
	C_2	272.126	105.263			
	A_3	92.000	221.000	C	272.18 ± 21.21	118.77 ± 19.443
	B_3	141.000	161.000			
	C_3	251.000	110.000			
**5**	A_1	63.235	220.946	A	71.27 ± 6.96	210.85 ± 13.22

	B_1	120.617	204.073			
	C_1	111.656	165.000			
	A_2	75.376	195.883	B	113.59 ± 6.08	175.32 ± 33.25
	B_2	109.742	138.914			
	C_2	105.340	99.631			
	A_3	75.205	215.736	C	110.09 ± 4.20	146.12 ± 40.50
	B_3	110.440	183.000			
	C_3	113.302	173.750			

A – kinetic parameters for pure enzyme (K_m, V_{max}); B – kinetic parameters of tested compounds (inhibitors) (1/3 of IC_{50} concentrations) ($K_{m(i)}$, $V_{max(i)}$); C - kinetic parameters of tested compounds (inhibitors) (IC_{50} concentrations) ($K_{m(i)}$, $V_{max(i)}$). Numerical index A_1, A_2 – the number of individual experiment (all experiments were conducted three times on different biological samples).

Table S2. *Kinetic parameters of BuChE enzymatic reactions.*

**COMPOUND**		**Km [μmol/L]**	**v_{max} [mA/min]**	**Mean ± SD**		
				**A. B. C**	**K_m [μmol/L]**	**v_{max} [mA/min]**
**1**	A_1	37.500	249.196	A	48.50 ± 12.04	240.49 ± 8.16
	B_1	122.244	223.809			
	C_1	291.683	178.705			
	A_2	62.039	239.275	B	109.28 ± 13.32	206.94 ± 15.02
	B_2	95.618	202.024			
	C_2	118.991	183.120			
	A_3	46.000	233.000	C	198.55 ± 87.14	174.27 ± 11.70
	B_3	110.000	195.000			
	C_3	185.000	161.000			
**2**	A_1	64.370	227.536	A	72.95 ± 8.40	232.85 ± 6.49
	B_1	129.816	178.466			
	C_1	229.370	81.110			
	A_2	73.322	230.936	B	118.59 ± 12.61	182.27 ± 9.29
	B_2	121.014	175.485			
	C_2	206.542	112.514			
	A_3	81.176	240.096	C	171.90 ± 80.57	103.11 ± 19.12
	B_3	104.941	192.871			
	C_3	79.806	115.727			
**3**	A_1	87.430	271.776	A	80.51 ± 11.73	264.63 ± 12.47
	B_1	61.128	207.848			
	C_1	103.901	166.268			

	A_2	87.144	271.902	B	88.23 ± 24.12	207.61 ± 5.30
	B_2	96.249	202.204			
	C_2	116.742	171.680			
	A_3	66.964	250.238	C	105.63 ± 10.35	180.05 ± 19.37
	B_3	107.337	212.802			
	C_3	96.249	202.204			
**4**	A_1	66.848	280.875	A	64.77 ± 14.37	240.87 ± 36.09
	B_1	78.523	249.675			
	C_1	75.748	180.395			
	A_2	49.473	176.753	B	69.78 ± 17.32	229.87 ± 19.38
	B_2	49.825	210.944			
	C_2	61.062	168.586			
	A_3	78.000	265.000	C	75.26 ± 13.97	174.32 ± 5.91
	B_3	81.000	229.000			
	C_3	89.000	174.000			
**5**	A_1	84.766	256.167	A	90.16 ± 6.13	276.25 ± 22.39
	B_1	94.621	229.384			
	C_1	101.124	200.088			
	A_2	88.888	300.400	B	104.42 ± 11.30	242.44 ± 12.47
	B_2	101.871	254.233			
	C_2	119.378	203.508			
	A_3	96.843	272.183	C	108.09 ± 1.39	202.78 ± 2.42
	B_3	116.795	243.730			
	C_3	103.778	204.771			

A – kinetic parameters for pure enzyme (K_m, V_{max}); B – kinetic parameters of tested compounds (inhibitors) (1/3 of IC_{50} concentrations) ($K_{m(i)}$, $V_{max(i)}$); C - kinetic parameters of tested compounds (inhibitors) (IC_{50} concentrations) ($K_{m(i)}$, $V_{max(i)}$). Numerical index A_1, A_2 – the number of individual experiment (all experiments were conducted three times on different biological samples).

Figure S1. The effects of substrate concentration on the enzymatic reaction. A) AChE and increasing concentration of acetyltiocholine iodide (ATC); B) BuChE and increasing concentration of butyryltiocholine iodide (BTC). The results are presented as mean ± SD of three independent experiments conducted in duplicates.

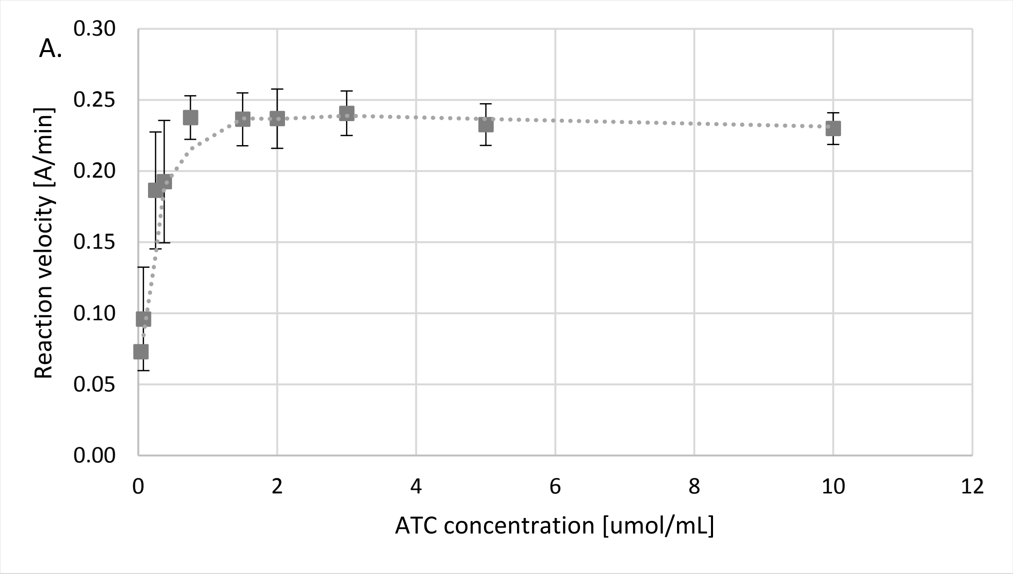

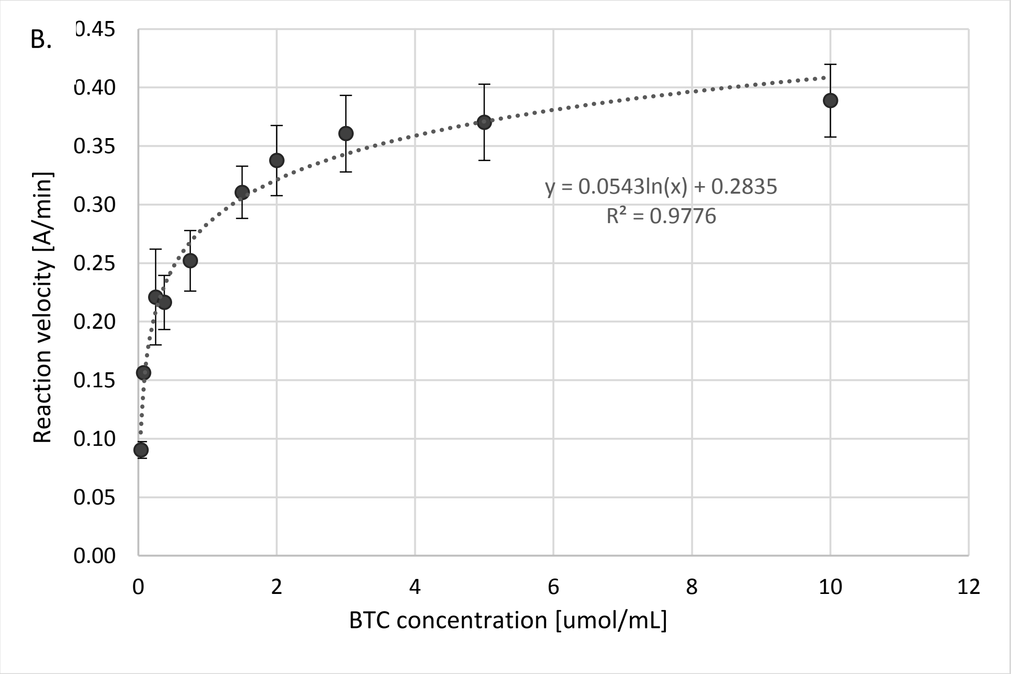

.
